# The effectiveness and safety of introducing condom-catheter uterine balloon tamponade for postpartum haemorrhage at secondary level hospitals in Uganda, Egypt and Senegal: a stepped wedge, cluster-randomised trial

**DOI:** 10.1111/1471-0528.15903

**Published:** 2019-09-18

**Authors:** HA Anger, R Dabash, J Durocher, N Hassanein, S Ononge, LJ Frye, A Diop, SB Beye, G Burkhardt, E Darwish, MC Ramadan, J Kayaga, D Charles, A Gaye, M Eckardt, B Winikoff

**Affiliations:** a Gynuity Health Projects, New York, NY, USA; b Obstetrician/Gynaecologist consultant, Alexandria, Egypt; c Makerere University School of Health Sciences, Kampala, Uganda; d Centre De Santé Philippe Senghor, Dakar, Senegal; e Alexandria Faculty of Medicine, Alexandria University, Alexandria, Egypt; f El Galaa Maternity Teaching Hospital, Cairo, Egypt; g Global Health Uganda, Kampala, Uganda; h Obstetrician/Gynaecologist, Dakar, Senegal; i Division of Global Health Innovation, Massachusetts General Hospital, Boston, MA, USA

**Keywords:** Maternal morbidity, maternal mortality, postpartum haemorrhage, refractory, treatment, uterine balloon tamponade

## Abstract

**Objective:**

To assess the effectiveness of introducing condom-catheter uterine balloon tamponade (UBT) for postpartum haemorrhage (PPH) management in low- and middle-income settings.

**Design:**

Stepped wedge, cluster-randomised trial.

**Setting:**

Eighteen secondary-level hospitals in Uganda, Egypt, and Senegal.

**Population:**

Women with vaginal delivery from October 2016 to March 2018.

**Methods:**

Use of condom-catheter UBT for PPH management was introduced using a half-day training and provision of pre-packaged UBT kits. Hospitals were randomised to when UBT was introduced. The incident rate (IR) of study outcomes was compared in the control (i.e. before UBT) and intervention (i.e. after UBT) periods. Mixed effects regression models accounted for clustering (random effect) and time period (fixed effect).

**Main outcome measures:**

Combined IR of PPH-related invasive surgery and/or maternal death.

**Results:**

There were 28 183 and 31 928 deliveries in the control and intervention periods, respectively. UBT was used for 9/1357 and 55/1037 women diagnosed with PPH in control and intervention periods, respectively. PPH-related surgery or maternal death occurred in 19 women in the control period (IR = 6.7/10 000 deliveries) and 37 in the intervention period (IR = 11.6/10 000 deliveries). The adjusted IR ratio was 4.08 (95% confidence interval 1.07–15.58). Secondary outcomes, including rates of transfer and blood transfusion, were similar in the trial periods.

**Conclusions:**

Introduction of condom-catheter UBT in these settings did not improve maternal outcomes and was associated with an increase in the combined incidence of PPH-related surgery and maternal death. The lack of demonstrated benefit of UBT introduction with respect to severe outcomes warrants reflection on its role.

**Tweetable abstract:**

Stepped wedge trial shows UBT introduction does not reduce the combined incidence of PPH-related surgery or death.

**Linked article:**

This article is commented on by AD Weeks, p. 1622 in this issue. To view this mini commentary visit https://doi.org/10.1111/1471-0528.15948.

## Introduction

Postpartum haemorrhage (PPH) remains the leading cause of maternal mortality.^1,2^Most diagnosed PPH is due to atony and is controlled by administration of uterotonics; however, some women continue to bleed and require interventions such as blood transfusion or surgery. In low- and middle-income countries (LMIC), which carry the heaviest burden of PPH-related mortality, these interventions are rarely available outside tertiary-level hospitals. Women may also experience delays in obtaining care due to absence of skilled surgical teams or blood shortages.^3,4^ To address this problem, there is growing interest in expanding use of uterine balloon tamponade (UBT), a method recommended by the World Health Organization.^5^

Observational and before-and-after studies from high-resource settings show a decline in invasive procedures for PPH (i.e. surgical intervention or uterine artery embolisation) associated with UBT introduction.^6–8^ Most research on UBT in high-resource settings has evaluated costly devices such as the Bakri balloon,^9,10^ but a similar effect can be achieved using the low-cost alternative of tying a condom to the end of a urinary catheter.^11,12^ A 2013 review of UBT in LMIC (mostly using condom-catheter devices) reported that bleeding was controlled after UBT for 234/241 (97.1%) women.^13^ Subsequently, two prospective, multi-country case series from LMIC assessed hundreds of UBT uses for refractory PPH and reported cessation of bleeding for 84–97% of women who received it.^14,15^ Although the high survival rates are encouraging, interpretation of the findings is complicated by lack of a comparison group.

Results from a randomised controlled trial of condom-catheter UBT conducted in Mali and Benin showed no difference in the primary composite outcome of invasive surgery and death among 116 women with refractory PPH randomised to receive UBT or misoprostol or misoprostol alone. Further, women who had UBT had higher rates of blood loss ≥1000 ml (*P* < 0.001) and maternal death (*P* = 0.059).^16^ Despite a relatively small sample size, these results highlighted the conundrum of the growing momentum for expanding UBT use in LMIC, despite no rigorous evidence showing a benefit of UBT introduction in LMIC health systems.^17^

To address these issues, we conducted a trial to assess whether introduction of UBT reduced overall PPH-related morbidity and mortality among hospital populations in three LMIC.

## Methods

This stepped wedge, cluster-randomised trial was conducted in 18 hospitals in Senegal, Egypt, and Uganda (six hospitals per country). This cluster-randomised design was chosen because our research question addressed whether facility-wide introduction of UBT led to reductions in PPH-related morbidity and mortality. Because UBT was hypothesised to be beneficial, we chose a stepped wedge design so that all sites eventually received the intervention. Ethical approval was obtained from relevant ethical review committees in the three countries. A waiver of individual informed consent was granted for collection of de-identified clinical data on women diagnosed with PPH.

Hospitals were eligible if they were secondary-level public hospitals, had an approximate weekly average of 160 vaginal deliveries, and agreed to integrate UBT into standard care in accordance with international guidelines. Most hospitals had surgical teams, though these were not always immediately available. The study population was women receiving care at study hospitals, with inclusion criteria of: (1) vaginal delivery; (2) delivery at a study hospital or referral to a study hospital for PPH after delivery elsewhere. Exclusion criteria included caesarean delivery, death before arrival to study hospitals, and transfer to another hospital before delivery. The study population was not involved in development of this research.

To ensure study periods were balanced, we stratified hospitals by country and delivery volume (categorised as high, medium, low by tertiles). Strata contained two to four facilities each (not all countries had all three categories of delivery volume). Facilities within strata were randomly assigned to intervention phase using a 1:1 allocation ratio. Randomisation was performed by Gynuity staff in New York. Hospital and research staff were blinded to assessment of hospital-wide outcomes until completion of the trial.

The study design staggered UBT introduction in phases. Because the primary outcome was expected to be rare, we maximised the length of phases and minimised the number of phases. Thus, this trial had three phases, each lasting 5 months:

*Baseline phase*: all sites commenced data collection;

*First intervention phase:* UBT introduced at nine sites and the remaining nine continued with pre-existing practices

*Second intervention phase*: UBT introduced at the remaining nine sites.

Each hospital had a control and intervention period.

The intervention was training and introduction of UBT into routine practice for refractory PPH. The training was developed by investigators from Massachusetts General Hospital, Gynuity, and local investigators. Using a training-of-trainers approach, we convened a master training with experienced obstetricians from each country, who then led the training at study hospitals. The half-day training provided a review of recommended management of PPH and instruction on UBT use for suspected atonic PPH uncontrolled by uterotonics. Contraindications for UBT were uterine rupture, retained placenta, and severe latex allergy. All cadres of clinicians who support labour and delivery care at study hospitals were trained. See Appendix S1 for more details on the training. The research team pre-packaged standardised UBT kits that contained these locally procured components: Foley catheter (at least 22-mm gauge), two to three condoms, two cotton strings, a 60-ml syringe, and catheter plug. Figure S1 shows a completed system. Kits were distributed to hospitals after the training. The study team conducted periodic visits to address questions and discuss cases of refractory PPH and UBT use.

On a weekly basis, designated study staff prospectively recorded the following aggregate data for vaginal deliveries: number of deliveries, number of women treated for PPH, number of blood transfusions for PPH, number of hysterectomies for PPH, and number of PPH-related maternal deaths. Study coordinators worked with treating providers to document the following individual-level information for women with PPH: demographics, obstetric history, labour course and delivery, time of PPH diagnosis, suspected cause of PPH, treatment given, difficulties encountered during management, and outcome. PPH diagnosis was defined as administration of interventions beyond prophylactic measures to control postpartum bleeding. For women who received UBT in the intervention period, additional information was collected on timing of UBT, problems with UBT, and bleeding condition after UBT. See Appendix S1 for additional information on data management.

The primary composite outcome was PPH-related maternal death and/or invasive procedures. Invasive procedures included any procedure requiring laparotomy (i.e. compression sutures, pelvic vessel ligation, repair of uterine rupture, hysterectomy). Secondary outcomes included the disaggregated incidence of PPH-related maternal death, hysterectomy, conservative surgery (i.e. surgery requiring laparotomy but not hysterectomy), incidence of blood transfusion for PPH, and transfer to another hospital after PPH diagnosis. Feasibility was assessed by examining reported problems related to UBT. Core outcome sets for PPH treatment were published after protocol development and were not used.^18^

We estimated that 43 200 vaginal deliveries would occur over the 15-month study period. This sample allowed detection of a 65% reduction in the primary outcome (80% power, α = 0.05, one-tailed test), assuming a rate of 0.4% before UBT introduction (based on previous studies of PPH mortality in Senegal and of UBT introduction).^6,19^

The formula described by Woertman et al.^20^ was used to calculate a 4.26 correction factor for the study design effect (assuming an intracluster correlation coefficient of 0.05). As a before-after study of UBT introduction showed a 74% reduction in invasive procedures,^6^ a detectable difference of 65% was considered reasonable.

To investigate potential differences in the control and intervention populations, we compared characteristics of women diagnosed with PPH using mixed effects logistic or linear regression for categorical and continuous variables, respectively, incorporating study site (i.e. cluster) as a random effect and time period as a fixed effect.

To test whether rates of primary and secondary outcomes differed in control and intervention periods, we calculated unadjusted incident rate ratios (IRR) and 95% confidence intervals (CI) using Poisson regression of weekly aggregate data on vaginal deliveries and outcomes. To calculate IRR and corresponding 95% CI adjusted for cluster and temporal trends, mixed effects Poisson regression was used incorporating study site as a random effect and study phase as a fixed effect.^21^ We assessed temporal trends by calculating incidence rates of the primary outcome over the three study phases and stratifying by intervention and control.

To understand how clinical characteristics (e.g. cause of PPH) and systems issues (e.g. supply shortages) contributed to severe outcomes, we conducted a *post hoc* analysis to compare these factors among women with PPH-related invasive procedures or death and women with PPH without these outcomes; mixed effects logistic regression incorporating study site as a random effect was used to test for statistically significant differences. We also conducted *post hoc* sensitivity analyses to determine whether results were impacted by: underlying temporal trends at outlier sites; inclusion of non-atonic causes of PPH in the outcomes; interactions between temporal trends and study site or country; the statistical model used. See Appendix S1 for details on sensitivity analyses. Analyses were specified *a priori* unless noted otherwise and were conducted in STATA 12 (StataCorp. 2011. *Stata Statistical Software: Release 12*. College Station, TX, USA).

An independent Data Safety Monitoring Board reviewed an interim analysis and safety data in November 2017.

This trial was funded by the Bill & Melinda Gates Foundation, who had no involvement in the conduct of the research or manuscript writing.

## Results

**Figure 1:**
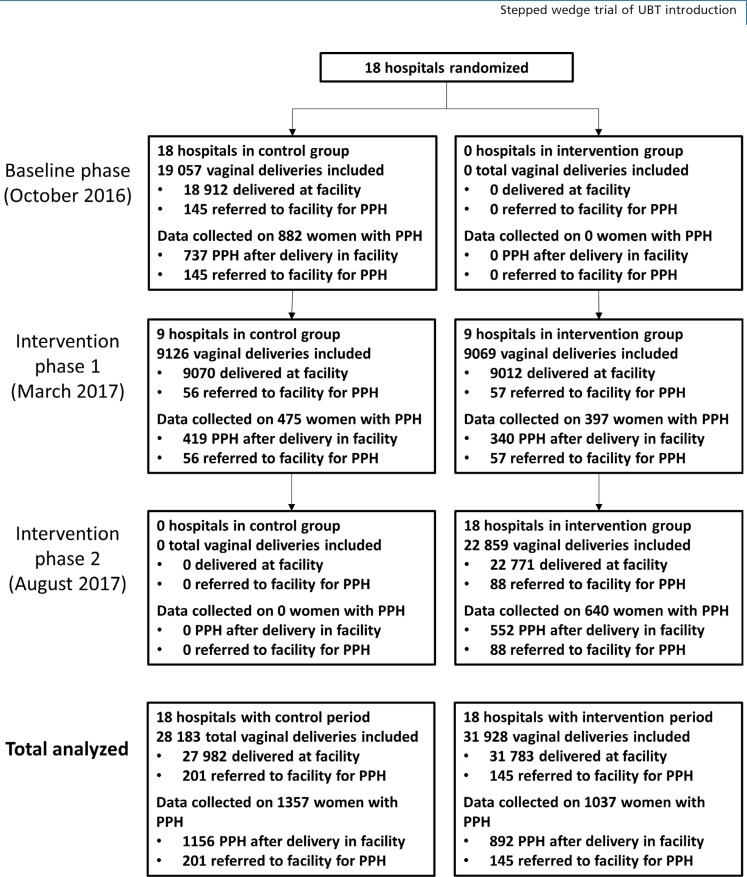
Trial profile. PPH, postpartum haemorrhage.

From October 2016 to March 2018, 59 765 vaginal deliveries occurred at study hospitals and 346 women were referred to 18 study hospitals for PPH after delivery elsewhere (Figure 1). Sites in Senegal contributed the most deliveries (*n* = 26 583), followed by Egypt (*n* = 17 181) and Uganda (*n* = 16 347). During control and intervention periods, there were 28 183 and 31 928 deliveries included in the analysis, respectively, and data were collected on 1357 (4.8%) and 1037 (3.3%) women diagnosed with PPH.

Study populations differed in control and intervention periods (Table 1). In the intervention period, significantly fewer women with PPH had labour augmentation (629 [49.3%] control versus 404 [42.8%] intervention) or retained placenta (228 [16.9%] control versus 156 [15.1%] intervention), and significantly more had atony (1046 [77.5%] control versus 862 [83.3%] intervention). Regarding PPH management, significantly fewer women in the intervention period received tranexamic acid (355 [49.3%] control versus 209 [42.8%] intervention), and more women in the intervention period had intravenous (IV) fluids (1273 [93.8%] control versus 982 [94.8%] intervention). Providers noted fewer shortages in supplies (170 [12.6%] control versus 100 [9.7%] intervention) or medications (206 [15.3%] control versus 146 [14.2%] intervention) in the intervention period.

**Table 1:** Characteristics of women diagnosed with PPH before and after UBT introduction

No. of PPH cases	Total 2394	Control period 1357	Intervention period 1037	*P*-value^*^
Age group (years) (%)				
<25	1109	643 (47.5)	466 (45.3)	Ref.
25–34	1005	555 (41.0	450 (43.8)	0.533
>35	268	156 (11.5)	112 (10.9)	0.889
**Parity (%)**				
Nulliparous	421	224 (16.5)	197 (19.0	Ref.
1–3	1519	877 (64.6)	642 (61.9)	0.944
>3	454	256 (18.9)	198 (19.1)	0.392
Referred to study facility for PPH	2394	201 (14.8)	145 (14.0)	0.10
**Procedures before delivery**				
(%) Induction	2227	176 (13.7)	93 (9.8)	0.10
Augmentation	2220	629 (49.3)	404 (42.8)	0.03
Stillbirth	2376	58 (4.3)	49 (4.8)	0.35
PPH prophylaxis—any uterotonic	2211	1166 (92.1)	918 (97.1)	0.05
**Time to PPH diagnosis**				
Minutes, mean (SD)	2343	53.2 (93.6)	51.1 (83.6)	0.19
Minutes, median (IQR)	2343	30 (50)	30 (30)	
≥1 hour after delivery	2343	236 (17.8%)	173 (17.1%)	0.04
**Suspected cause of PPH (multiple causes may be noted for one woman) (%)**				
Atony	2384	1046 (77.5)	862 (83.3)	0.04
Atony alone (no other cause noted)	2384	700 (51.9)	585 (56.5)	<0.01
Traumatic cause	2384	451 (33.4)	342 (33.0)	0.15
Retained placenta	2384	228 (16.9)	156 (15.1)	<0.01
**PPH interventions (%)**				
≥1 uterotonic	2388	1324 (97.9)	1027 (99.2)	0.42
>1 uterotonic	2388	996 (73.6)	825 (79.7)	0.12
Tranexamic acid	2383	355 (26.3)	209 (20.2)	0.03
Manual exploration/clot removal	2392	1190 (87.7)	836 (80.8)	0.84
Intravenous fluids	2393	1273 (93.8)	982 (94.8)	0.01
Manual removal of placenta	2393	222 (16.4)	162 (15.6)	<0.01
Suturing lacerations or tears	2392	540 (39.8)	395 (38.2)	0.12
Bimanual compression	2392	573 (42.2)	490 (47.3)	0.11
UBT	2394	9 (0.7)	55 (5.3)**	<0.01
Problems reported by providers during course of PPH treatment (%)				
Supplies not available	2378	170 (12.6)	100 (9.7)	<0.01
Medication not available	2378	206 (15.3)	146 (14.2)	<0.01
No blood/insufficient blood available	2378	62 (4.6)	60 (5.8)	0.40
Necessary personnel not available	2378	11 (0.8)	4 (0.4)	NA**
Delays obtaining patient/family consent for procedure	2378	9 (0.7)	16 (1.6)	0.41

IQR, interquartile range; PPH, postpartum haemorrhage; SD, standard deviation; UBT, uterine balloon tamponade.

* *P*-values derived from mixed effects logistic regression models for categorical variables and mixed effects linear regression for continuous variables; mixed effects models adjust for study time period (fixed effect) and cluster (random effect).

** Not applicable: mixed effects logistic regression model would not converge due to small numbers.

### Outcomes

Uterine balloon tamponade introduction was associated with a statistically significant increase in the composite outcome of PPH-related invasive procedures and/or maternal death (Table 2). There were 19 events among 28 183 deliveries in the control period (6.7/10 000 deliveries) and 37 events among 31 928 deliveries in the intervention period (11.6/10 000 deliveries, unadjusted IRR = 1.72, 95% CI 0.99–2.99). In the adjusted model, the IRR rose to 4.08 (95% CI 1.07–15.58). Temporal trends of the primary outcome show that sites randomised to introduce UBT in the first intervention phase had an increase in the rate during intervention phase 1 (when UBT was introduced) compared with baseline, and the rate fell to near baseline levels in intervention phase 2. Sites randomised to intervention phase 2 had a decline in the rate from baseline phase to intervention phase 1, followed by an increase in intervention phase 2 when UBT was introduced (Table S1).

**Table 2:** Outcomes of the stepped wedge cluster-randomised trial

	Control period	Intervention period	Unadjusted model		Mixed effects model (adjusted for study design)	
	*n* (per 10 000 deliveries)	*n* (per 10 000 deliveries)	IRR (95% CI)^a^	*P*-value^a^	IRR (95% CI)^b^	*P*-value^b^
Total, *n*	28 183	31 928				
**Primary outcome**						
Maternal death due to PPH or invasive procedures for PPH^c^	19 (6.7)	37 (11.6)	1.72 (0.99–2.99)	0.06	4.08 (1.07–15.58)	0.04
**Secondary outcomes**						
Maternal death due to PPH	10 (3.5)	15 (4.7)	1.32 (0.59–2.95)	0.49	2.23 (0.35–14.07)	0.39
Hysterectomy due to PPH	7 (2.5)	13 (4.1)	1.64 (0.65–4.11)	0.29	4.38 (0.47–41.09)	0.20
Conservative surgery for PPH^d^	5 (1.8)	16 (5.1)	2.82 (1.03–7.71)	0.04	Cannot estimate^e^	
Blood transfusion for PPH	282 (100.1)	311 (97.4)	0.97 (0.83–1.14)	0.74	1.24 (0.86–1.80)	0.25
Transfer to higher level care after PPH diagnosis	21 (7.5)	16 (5.0)	0.67 (0.35–1.29)	0.23	3.05 (0.79–11.70	0.10

CI, confidence interval; IRR, incident rate ratio; PPH, postpartum haemorrhage.

a Derived from simple Poisson regression models.

b Derived from mixed effects models include study site (cluster) as a random effect and study time period as a fixed effect.

c Invasive procedures defined as hysterectomy or conservative surgical procedures (includes arterial ligation, B Lynch/compression sutures, repairof ruptured uterus).

d Surgical intervention for PPH that requires laparotomy but excludes hysterectomy (includes arterial ligation, B Lynch/compression sutures, repair of ruptured uterus).

e Mixed effects Poisson regression model could not generate an estimate as there were no conservative surgical procedures observed among sites that did not start UBT in the first intervention phase.

From the control to intervention periods, there were slight increases in rates of maternal death (3.5/10 000 versus 4.7/10 000) and hysterectomy (2.5/10 000 versus 4.1/10 000); these differences were not statistically significant (Table 2). There was an increase in the use of conservative surgical procedures in unadjusted analysis (1.8/10 000 versus 5.0/10 000, crude IRR = 2.82, 95% CI 1.03–7.71), although we could not generate an adjusted estimate due to small numbers. There were no notable trends in blood transfusion or transfer to another hospital.

In sensitivity analyses (i.e. excluding outlier hospitals, restricting analysis to outcomes associated with atonic PPH, adjusting for interaction of temporal trends by site or country), outcome trends remained the same, although the difference in the primary outcome was not statistically significant (Figure S2, Tables S2–S4).

### UBT use

**Figure 2:**
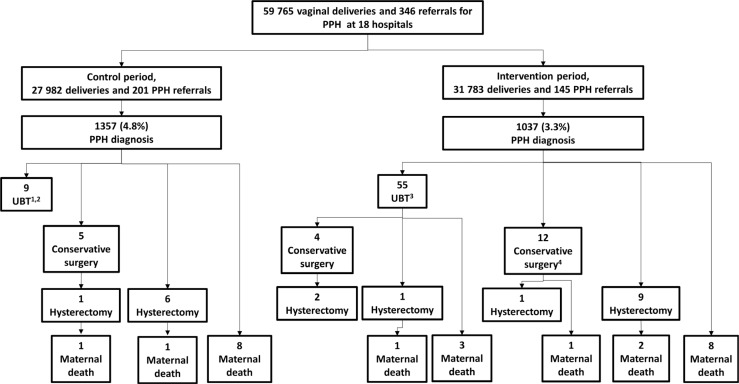
Outcomes for the stepped wedge cluster-randomised trial of UBT introduction in Uganda, Egypt, and Senegal, October 2016 to March 2018. PPH, postpartum haemorrhage; UBT, uterine balloon tamponade. ^1^Providers at four sites were independently using UBT before study UBT training and introduction (improvised kits containing catheter with condom or glove). ^2^Nine women who had UBT before study UBT training had bleeding controlled without need for surgical intervention. ^3^Of 55 women who had UBT after study UBT training, 47 had bleeding controlled without need for surgical intervention. ^4^Includes one woman for whom providers attempted to use UBT but a part was missing when assembling, so they proceeded to surgery.

In the control period, nine women received UBT because individual providers from four sites were already independently using UBT; there was no invasive surgery or maternal death in any women (Figure 2).

Fifty-five women received UBT in the intervention period. Five (9.1%) were referred to study facilities for PPH. All 55 women received uterotonics before UBT. UBT was used a median of 30 minutes (range 0–510 minutes) after PPH diagnosis. Providers reported a problem with UBT use in 25/48 (52.1%) cases (information was missing for seven women). The most common problems were more than one attempt before successful insertion (*n* = 15) and balloon displacement (*n* = 10). Providers reported that bleeding was controlled after UBT for 44/55 (80.0%). Ultimately, 47/55 (85.5%) women who received UBT recovered without invasive surgery, two (3.6%) had conservative surgery for PPH without further intervention, two (3.6%) had conservative surgery and then hysterectomy, one (1.8%) had hysterectomy and died, and three (5.5%) died without receiving surgery. Appendix S1 contains more details on women who died after receiving UBT. Notably, 29/37 (78.4%) women who had PPH-related surgery or maternal death in the intervention period did not receive UBT (Figure 2).

### Factors associated with invasive surgery or maternal death

Table 3 shows notable characteristics of women with PPH-related invasive surgery and/or death. When compared with 2338 women who recovered from PPH without invasive procedures, the 56 women who died or had surgery were more likely to be referred to the hospital for PPH (19/56 [33.9%] versus 327/2338 [14.0%]), have a traumatic cause of PPH (33/54 [61.1%] versus 760/2330 [32.6%]) or have providers report a blood shortage (15/53 [28.3%] versus 107/2325 [4.6%]) or that necessary health personnel were unavailable (5 [9.4%] versus 10 [0.4%]).

**Table 3:** Characteristics of women with PPH who did or did not have invasive surgery or maternal death due to PPH

	No maternal death or invasive surgery	Maternal death or invasive surgery	*P*-value*
No. of women	2338	56	
Referred to study facility for PPH	327 (14.0%)	19 (33.9%)	0.04
**Time to PPH diagnosis**	*n* = 2295	*n* =48	
Minutes, mean (SD) [*n*]	52.2 (89.5)	55.1 (84.2)	0.61
Minutes, median (IQR) [*n*]	30 (45)	30 (45)	
≥1 hour after delivery	398 (17.3%)	11 (22.9%)	0.74
**Suspected cause of PPH**	*n* = 2330	*n* = 54	
Atony	1869 (80.2%)	39 (72.2%)	0.28
Atony alone (no other cause noted)	1267 (54.8%)	18 (33.3%)	0.01
Traumatic cause	760 (32.6%)	33 (61.1%)	<0.01
Retained placenta	375 (16.1%)	9 (16.7%)	0.79
Any non-atonic cause	1025 (44.0%)	36 (66.7%)	< 0.01
**Problems reported by providers during course of PPH treatment**	*n* = 2325	*n* = 53	
Supplies not available	261 (11.2%)	9 (17.0%)	0.50
Medication not available	347 (14.9%)	5 (9.4%)	0.03
No blood/insufficient blood available	107 (4.6%)	15 (28.3%)	<0.01
Necessary personnel not available	10 (0.4%)	5 (9.4%)	<0.01
Delays obtaining patient/family consent for procedure	23 (1.0%)	2 (3.8%)	0.07

IQR, interquartile range; PPH, postpartum haemorrhage; SD, standard deviation.

* *P*-values derived from mixed effects logistic regression models for categorical variables and mixed effects linear regression for continuous variables; mixed effects models adjust for study time period cluster (random effect).

Regarding the 25 PPH-related deaths, 8/25 (32.0%) were referred to hospitals for PPH, 13/23 (56.5%) had delivered a stillbirth, and 2/24 (8.3%) were diagnosed with uterine rupture. Atony was noted in 18/24 (72.0%), though traumatic causes were also common (11/24, 45.8%). PPH management included uterotonics (22/24, 91.7%), blood transfusion (15/25, 60.0%) and/or surgical intervention (6/25, 24.0%). Blood shortages were common (11/23, 47.8%).

## Discussion

### Main findings

This stepped wedge, cluster-randomised trial showed an increase in the composite outcome of PPH-related invasive procedures and/or maternal death following UBT introduction at secondary-level hospitals in Uganda, Egypt, and Senegal. Underlying temporal trends in the primary outcome may account for the observed increase.

### Strengths and limitations

This is the first report of a study assessing the effectiveness of UBT introduction on reducing overall PPH-related morbidity and mortality among hospital populations in LMIC using a stepped-wedge, cluster-randomised trial design. Prior to this research, evidence on UBT use in LMIC was largely comprised of case series with a key limitation of no comparison group. Our study was designed to address this evidence gap and to better understand the specific impact that UBT introduction may have as a public health intervention aimed at improving PPH-related outcomes in LMIC.

An important limitation of this study is the complexity of the composite outcome of PPH-related invasive surgery and/or death. A pronounced increase in conservative surgical procedures at one site drove the overall increase in the primary outcome rate observed among hospitals that started UBT in the first intervention phase, whereas sites that did not introduce UBT in that phase had a decrease. These diverging trends explain the difference in crude and adjusted analysis, as the latter adjusted for temporal trends. We believe temporal trends acted as a confounder in this analysis and that UBT did not play a direct role in the increase in the primary outcome, as most women (78.4%) with invasive surgery or death in the intervention period did not receive UBT. However, numerous sensitivity analyses did not change the study trends; thus, it is possible that UBT introduction played an *indirect* role in the increase. For example, increased use of surgery may have been an unintended consequence of the trial’s UBT training, which focused on refractory haemorrhage and emphasised the need for second-line interventions when uterotonics do not control bleeding. We are also aware of one independent seminar on surgical management of PPH that occurred in Egypt near the beginning of the first intervention period (though this seminar was not held at or near study hospitals). Notably, the increases in use of UBT and surgery in intervention periods was accompanied by a slight but not statistically significant increase in PPH-related mortality. Dumont et al.^16^ posited that UBT could contribute to worse outcomes by introducing an extra step in the care pathway and delaying surgery. In our study, most women who died in the intervention period did not receive UBT, thus it is unlikely that UBT-associated delays contributed to this modest rise in deaths.

Another limitation is that participants were not individually randomised, thus it is uncertain whether populations in the control and intervention periods were comparable. Women with PPH in the two periods differed in some factors, though differences seemed to favour improved outcomes in the UBT intervention period (e.g. larger proportion of atonic PPH, fewer reports of supply or medication shortages). Also, it is difficult to draw conclusions about the effect of UBT, as it was so rarely used after introduction (0.17% of deliveries). This reflects the rarity of refractory atonic PPH at these secondary hospitals. Thirdly, providers reported a problem in half of UBT uses, which could reflect typical problems associated with a new technology, but could also hamper the willingness of providers to adopt the method. Finally, our study duration may have been too short to allow health providers time to gain confidence with UBT and to observe benefits of UBT introduction.

### Interpretation

Women who received UBT in our study had a high survival rate (92.7%), which was similar to survival rates of 94.5–97.4% reported in previous case series assessing use of condom-catheter UBT in LMIC^14,15^ These results suggest that UBT may be an effective intervention for some women. Nevertheless, our study’s primary analysis showed an overall increase in PPH-related death and/or invasive procedures following UBT introduction.

It is concerning that UBT, an intervention widely considered to be effective, did not show a benefit on overall outcomes based on results from this study and of a previous trial of condom-catheter UBT conducted in Benin and Mali by Dumont et al.^16^ Further, results of both studies suggest possible harm. In contrast, studies conducted in high-resource settings show that UBT introduction was associated with a decline in invasive procedures for PPH.^6–8^ These conflicting findings may be explained by differences in study contexts, as both our study and the Dumont trial were conducted in LMIC where the clinical benefit of UBT may be attenuated by the lack of system capacity to address obstetric emergencies such as refractory PPH adequately. For example, blood shortages were a problem for almost half of PPH-related deaths in our study; in some cases, bleeding stopped after UBT, yet women did not recover because timely blood replacement was unavailable. These findings suggest that interventions such as UBT may have limited effectiveness in improving maternal outcomes when introduced into resource-constrained health systems with unreliable access to other essential components of emergency care. Because the management of refractory haemorrhage requires a response that is complex and is thus dependent on other health system capacities, it is difficult to judge the effect of UBT introduction and to generalise our study findings. More encouraging results of UBT implementation may be observed elsewhere with more favourable environments (e.g. reliable blood supply), with a different UBT device or with a longer observation period.

Another reason why this study did not show a benefit of UBT introduction was the role of non-atonic causes in refractory PPH. As UBT is designed for treatment of atonic PPH and because atony plays a role in most diagnosed PPH, we hypothesised that introduction of UBT would address most refractory PPH and lead to declines in overall PPH-related morbidity and mortality. In our study, approximately 80.0% of diagnosed PPH was due to atony; however, 66.7% of women with PPH-related invasive surgery or death had PPH complicated by non-atonic causes. Similarly, Shakur et al.^22^ documented that 51.6% of PPH cases who had hysterectomy or died had non-atonic causes,^22^ and Mousa et al.^23^ reported that 50% of women with PPH unresponsive to first-line therapy had traumatic causes.^23^ These findings suggest that most atonic PPH can be effectively treated with first-line interventions and that refractory PPH may require different resources.

## Conclusion

The increase in severe maternal outcomes associated with UBT introduction in this trial, though likely driven by unrelated temporal trends, warrants careful consideration on the role of UBT within maternal health strategies in LMIC. Further research in LMIC is needed to help refine protocols for managing refractory haemorrhage and ensure that new approaches do no harm. In the end, the problem of PPH morbidity and mortality is multi-causal and complex, and it is likely that more than a single intervention is needed to show a discernible improvement in maternal outcomes in LMIC. To ensure that efforts lead to tangible reductions in maternal mortality and morbidity, future approaches should prioritise the strengthening of health systems, including addressing personnel shortages and unreliable blood supply.

## Supporting Information

**Figure S1.** Example of a completed condom-catheter UBT system used in the trial.

**Figure S2.** Sensitivity analysis by site: estimated incident rate ratios (IRR) after excluding each site from mixed effects Poisson regression models.

**Table S1.** Incidence rates of the primary outcome (PPH-related invasive surgery or death) by study period and when sites introduced UBT.

**Table S2.** Unadjusted and adjusted analysis of primary and secondary outcomes after excluding two sites identified as outliers (see Figure S2).

**Table S3.** Sensitivity analysis restricting outcomes to only measured among women with atonic PPH (including where atony is the only cause noted, or where atony is noted in presence of other causes of PPH).

**Table S4.** Sensitivity analysis using different statistical models to adjust for study design and inclusion of interaction terms.

**Appendix S1.** Supplementary methods and results.
