# Supplementary material for: The effectiveness and safety of introducing condom-catheter uterine balloon tamponade for postpartum haemorrhage at secondary level hospitals in Uganda, Egypt and Senegal: a stepped wedge, cluster-randomised trial
Source: BJOG. 2019 Sep 18;126(13):1612–21. doi: 10.1111/1471-0528.15903 (PMC6899652; doi:10.1111/1471-0528.15903)
Supplement: Supplementary file 1 [file BJOG-2019-1471-0528-15903-s1.docx]

**Table S1.** Incidence rates of the primary outcome (PPH-related invasive surgery or death) by study period and when sites introduced UBT.

|  | **Sites randomized to Step 1** | | | **Sites randomized to Step 2** | | |
| --- | --- | --- | --- | --- | --- | --- |
|  | **Phase** | **N vaginal deliveries** | **N events (per 10,000 deliveries)** | **Phase** | **N vaginal deliveries** | **N events (per 10,000 deliveries)** |
| Baseline period | Control | 9580 | 7 (7.3) | Control | 9477 | 9 (9.5) |
| Intervention step 1 | Intervention | 9069 | 15 (16.5) | Control | 9126 | 3 (3.3) |
| Intervention step 2 | Intervention | 11,776 | 13 (11.0) | Intervention | 11,083 | 9 (8.1) |

**Table S2.** Unadjusted and adjusted analysis of primary and secondary outcomes after excluding two sites identified as outliers (see Figure 2s).

|  | **Control period,** | **Intervention period,** | **Unadjusted model^a^** | | **Mixed effects Poisson regression model^b^** | |
| --- | --- | --- | --- | --- | --- | --- |
|  | **N (per 10,000 deliveries)** | **N (per 10,000 deliveries)** | **IRR (95% CI)** | **P value** | **IRR (95% CI)** | **P value** |
| Total, N | 25,116 | 28,092 |  |  |  |  |
| **Primary outcome** |  |  |  |  |  |  |
| Maternal death due to PPH or invasive procedures for PPH^c^ | 13 (4.6) | 20 (6.3) | 1.38 (0.68-2.77) | 0.37 | 4.50 (0.48-42.11) | 0.19 |
| **Secondary outcomes** |  |  |  |  |  |  |
| Maternal death due to PPH | 6 (2.1) | 11 (3.4) | 1.64 (0.61-4.43) | 0.33 | Cannot estimate^e^ |  |
| Hysterectomy due to PPH | 6 (2.1) | 8 (2.5) | 1.19 (0.41-3.44) | 0.75 | 1.97 (0.16-24.0) | 0.59 |
| Conservative surgery for PPH^d^ | 2 (0.7) | 3 (0.9) | 1.34 (0.22-8.03) | 0.75 | Cannot estimate^e^ |  |
| Blood transfusion for PPH | 157 (55.7) | 223 (69.8) | 1.27 (1.04-1.56) | 0.02 | 1.38 (0.88-1.16) | 0.16 |
| Transfer to higher level care after PPH diagnosis | 19 (6.7) | 16 (5.0) | 0.75 (0.39-1.46) | 0.40 | 3.79 (0.91-15.79) | 0.07 |

Abbreviations: IRR=incident rate ratio, CI=confidence interval, PPH=postpartum hemorrhage ^a^Derived from simple Poisson regression models, ^b^Derived from mixed effects models include study site (cluster) as a random effect and study time period as a fixed effect, ^c^Invasive procedures defined as hysterectomy or conservative surgical procedures (includes arterial ligation, B Lynch/compression sutures, repair of ruptured uterus), ^d^Surgical intervention for PPH that requires laparotomy, but excludes hysterectomy (includes arterial ligation, B Lynch/compression sutures, repair of ruptured uterus). ^e^Mixed effects Poisson regression model could not generate an estimate due to small number of events over subtrata.

**Table S3.** Sensitivity analysis restricting outcomes to only measured among women with atonic PPH (including where atony is the only cause noted, or where atony is noted in presence of other causes of PPH).

|  | **Control period,** | **Intervention period,** | **Unadjusted model ^a^** | | **Mixed effects model (adjusted for study design)^b^** | |
| --- | --- | --- | --- | --- | --- | --- |
|  | **N (per 10,000 deliveries)** | **N (per 10,000 deliveries)** | **IRR (95% CI)** | **P value** | **IRR (95% CI)** | **P value** |
| Total, N | 28183 | 31928 |  |  |  |  |
| **PPH OUTCOMES DUE TO ATONY ALONE** |  |  |  |  |  |  |
| **Primary outcome** |  |  |  |  |  |  |
| Maternal death due to PPH or invasive procedures for PPH^c^ | 7 (2.5) | 11 (3.4) | 1.39 (0.54-3.58) | 0.50 | 1.53 (0.25-9.43) | 0.64 |
| **Secondary outcomes** |  |  |  |  |  |  |
| Maternal death due to PPH | 5 (1.8) | 7 (2.2) | 1.24 (0.39-3.89) | 0.72 | 2.11 (0.18-24.98) | 0.55 |
| Hysterectomy due to PPH | 2 (0.7) | 3 (0.9) | 1.32 (0.22-7.92) | 0.76 | CE^g^ |  |
| Conservative surgery for PPH^f^ | 0 (0) | 5 (1.6) | CE^g^ |  | CE^g^ |  |
| Blood transfusion for PPH | 131 (46.5) | 145 (45.4) | 0.97 (0.77-1.24) | 0.85 | 1.78 (1.04-3.05) | 0.04 |
| Transfer to higher level care after PPH diagnosis | 5 (1.8) | 6 (1.9) | 1.06 (0.32-3.47) | 0.23 | 4.12 (0.32-52.38) | 0.28 |
| **PPH OUTCOMES DUE TO ATONY (with or without other cause)** |  |  |  |  |  |  |
| **Primary outcome** |  |  |  |  |  |  |
| Maternal death due to PPH or invasive procedures for PPH^e^ | 14 (5.0) | 25 (7.8) | 1.57 (0.82-3.03) | 0.17 | 2.94 (0.71-12.27) | 0.14 |
| **Secondary outcomes** |  |  |  |  |  |  |
| Maternal death due to PPH | 8 (2.8) | 10 (3.1) | 1.10 (0.44-2.80) | 0.84 | 1.72 (0.24-12.17) | 0.59 |
| Hysterectomy due to PPH | 4 (1.4) | 8 (2.5) | 1.77 (0.53-5.83) | 0.35 | 1.80 (0.15-21.53) | 0.64 |
| Conservative surgery for PPH^f^ | 4 (1.4) | 13 (4.1) | 2.87 (0.94-8.80) | 0.07 | CE^g^ | 0.99 |
| Blood transfusion for PPH | 211 (74.9) | 266 (83.3) | 1.11 (0.93-1.33) | 0.25 | 1.49 (0.98-2.27) | 0.07 |
| Transfer to higher level care after PPH diagnosis | 13 (4.6) | 10 (3.1) | 0.68 (0.30-1.55) | 0.36 | 2.33 (0.47-11.61) | 0.30 |

Abbreviations: PPH=postpartum hemorrhage, IRR=incident rate ratio, CE=cannot estimate. ^a^ Derived from simple Poisson regression models, ^b^ Derived from mixed effects models include study site (cluster) as a random effect and study time period as a fixed effect, ^c^Invasive procedures defined as hysterectomy or conservative surgical procedures (includes arterial ligation, B Lynch/compression sutures, repair of ruptured uterus), ^d^Surgical intervention for PPH that requires laparotomy, but excludes hysterectomy (includes arterial ligation, B Lynch/compression sutures, repair of ruptured uterus). ^g^Mixed effects Poisson regression model could not generate an estimate due to small number of events over subtrata.

**Table S4.** Sensitivity analysis using different statistical models to adjust for study design and inclusion of interaction terms.

|  | **Control period** | **Intervention period** | **Unadjusted model^a^** | | **Poisson regression with GEE^b^** | | **Negative binomial regression with GEE^c^** | | **Mixed effects Poisson regression with country and time period interaction term^d^** | | **Mixed effects Poisson regression, allow temporal trends to vary by cluster^e^** | |
| --- | --- | --- | --- | --- | --- | --- | --- | --- | --- | --- | --- | --- |
|  | N **(per 10,000 deliveries)** | N **(per 10,000 deliveries)** | **IRR (95% CI)** | **P value** | **IRR (95% CI)** | **P value** | **IRR (95% CI)** | **P value** | **IRR (95% CI)** | **P value** | **IRR (95% CI)** | **P value** |
| Total, N | 28183 | 31928 |  |  |  |  |  |  |  |  |  |  |
| **Primary outcome** |  |  |  |  |  |  |  |  |  |  |  |  |
| Maternal death due to PPH or invasive procedures for PPH^e^ | 19 (6.7) | 37 (11.6) | 1.72 (0.99-2.99) | 0.06 | 5.08 (1.02-25.17) | 0.04 | 4.97 (1.00-24.70) | 0.05 | 3.65 (0.94-14.25) | 0.06 | 4.00 (0.99-16.13) | 0.05 |
| **Secondary outcomes** |  |  |  |  |  |  |  |  |  |  |  |  |
| Maternal death due to PPH | 10 (3.5) | 15 (4.7) | 1.32 (0.59-2.95) | 0.49 | 2.61 (0.25-27.02) | 0.42 | 2.61 (0.25-26.89) | 0.42 | CE |  | 2.23 (0.35-14.03) | 0.39 |
| Hysterectomy due to PPH | 7 (2.5) | 13 (4.1) | 1.64 (0.65-4.11) | 0.29 | 4.54 (0.55-37.05) | 0.16 | 4.49 (0.55-36.71) | 0.16 | CE |  | 4.35 (0.46-40.88) | 0.20 |
| Conservative surgery for PPH^f^ | 5 (1.8) | 16 (5.1) | 2.82 (1.03-7.71) | 0.04 | CE |  | CE |  | CE |  | CE |  |
| Blood transfusion for PPH | 282 (100.1) | 311 (97.4) | 0.97 (0.83-1.14) | 0.74 | 1.30 (0.45-3.77_ | 0.63 | 1.26 (0.58-2.76) | 0.56 | 1.35 (0.93-1.99) | 0.12 | 1.39 (0.70-2.76) | 0.35 |
| Transfer to higher level care after PPH diagnosis | 21 (7.5) | 16 (5.0) | 0.67 (0.35-1.29) | 0.23 | 2.39 (0.56-10.11) | 0.24 | 2.38 (0.56-10.00) | 0.24 | 3.81 (0.84-17.21) | 0.08 | 3.01 (0.76-11.94) | 0.12 |

Abbreviations: GEE=generalized estimating equations, IRR=Incident rate ratio, CI=confidence interval, PPH=postpartum hemorrhage, CE=Cannot estimate due to small number of events over substrata. ^a^Simple Poisson regression model modeling crude estimate of treatment effect, ^b^ Poisson regression model adjusting for study time period (fixed effect) and invoking generalized estimating equations with sandwich estimator to adjust for cluster, ^c^Negative binomial regression model adjusting for study time period (fixed effect) and invoking generalized estimating equations with sandwich estimator to adjust for cluster, ^d^Mixed effects Poisson regression model adjusting for cluster (random effect), study time period (fixed effect) and inclusion of an interaction term between study time period and country (fixed effect), ^e^Mixed effects Poisson regression model adjusting for cluster (random effect) and study time period (fixed effect) and allowing the estimated slopes of the treatment effect to vary by cluster
