# Supplementary figures and images for: The effectiveness and safety of introducing condom-catheter uterine balloon tamponade for postpartum haemorrhage at secondary level hospitals in Uganda, Egypt and Senegal: a stepped wedge, cluster-randomised trial
Source: BJOG. 2019 Sep 18;126(13):1612–21. doi: 10.1111/1471-0528.15903 (PMC6899652; doi:10.1111/1471-0528.15903)

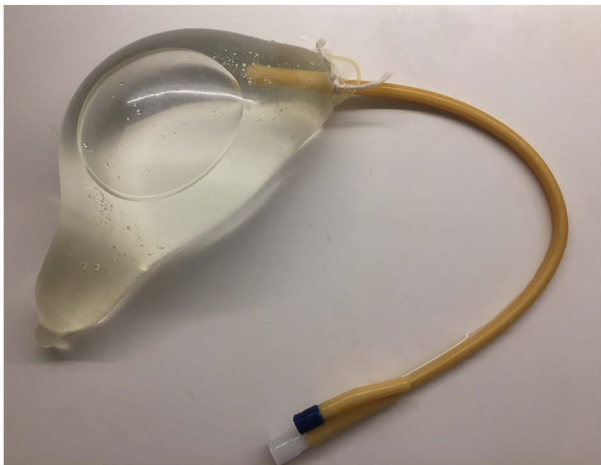

**Figure S1.** Example of a completed condom-catheter UBT system used in the trial.

Supplement: Supplementary file 2 [file BJOG-2019-1471-0528-15903-s2.pdf]
