# Supplementary material for: The effectiveness and safety of introducing condom-catheter uterine balloon tamponade for postpartum haemorrhage at secondary level hospitals in Uganda, Egypt and Senegal: a stepped wedge, cluster-randomised trial
Source: BJOG. 2019 Sep 18;126(13):1612–21. doi: 10.1111/1471-0528.15903 (PMC6899652; doi:10.1111/1471-0528.15903)
Supplement: Supplementary file 3 [file BJOG-2019-1471-0528-15903-s3.pdf]

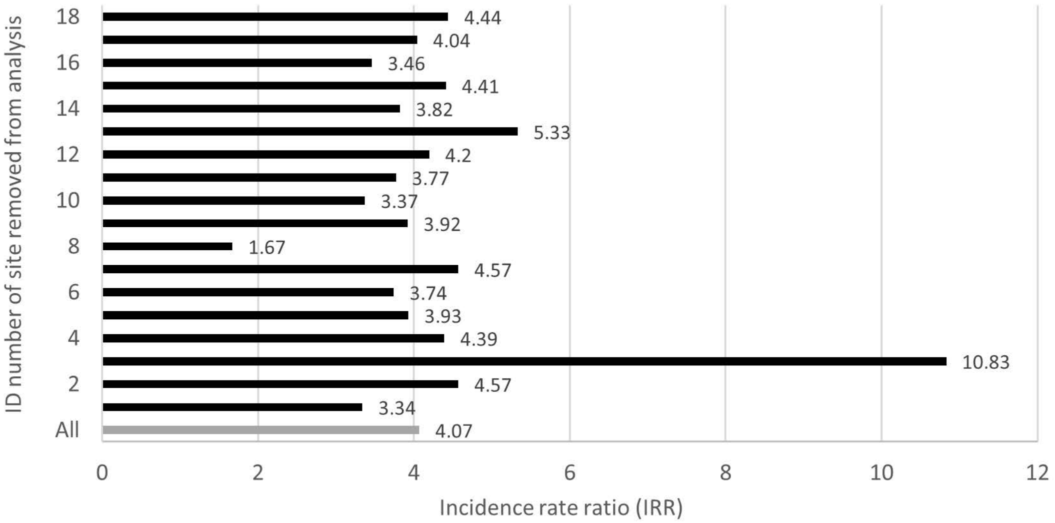

Figure S2. Sensitivity analysis by site: estimated incident rate ratios (IRRs) after excluding each site from mixed effects Poisson regression models. Site 3 and Site 8 were identified as outlier sites and excluded in a sensitivity analysis of primary and secondary outcomes (see Table S1).
