# Supplementary material for: The effectiveness and safety of introducing condom-catheter uterine balloon tamponade for postpartum haemorrhage at secondary level hospitals in Uganda, Egypt and Senegal: a stepped wedge, cluster-randomised trial
Source: BJOG. 2019 Sep 18;126(13):1612–21. doi: 10.1111/1471-0528.15903 (PMC6899652; doi:10.1111/1471-0528.15903)
Supplement: Supplementary file 4 [file BJOG-2019-1471-0528-15903-s4.pdf]

Appendix S1. Supplementary methods and results

Supplementary methods

Additional information on the study training

The training course was developed in collaboration with an expert from Massachusetts General Hospital (ME) who helped develop the training approach used in several published multi-country case series that showed encouraging results of uterine balloon tamponade (UBT).[1,2] The training approach used in this present study was based on the model used in the published cases which were all conducted in low- and middle-income settings; thus, it was believed that this was the most evidence-based training approach that could be applied. The training was a comprehensive review of non-surgical management of PPH along with use of UBT with pre-assembled kits for refractory PPH. The didactic portion of the training on UBT included indications and contraindications for use, instruction on device assembly, monitoring the woman after balloon insertion, and trouble-shooting. Practical training included hands-on skills sessions on assembly of the balloon, as well as insertion, placement, and inflation of the balloon using simple uterine models (water bottle wrapped in a pillow). The training specified that one condom should be tied with the two cotton strings at the insertion end such that the catheter balloon is inside the condom. The condom balloon should be filled with clean water (i.e. clean tap water was acceptable). The extra condoms were included in the kit in case one was dropped or otherwise mishandled. All cadres of clinicians who support labor and delivery care at study hospitals were trained. Local clinicians at study sites may have had some previous knowledge of UBT, but the majority had no previous direct experience using UBT.

In general, the training first reviewed elements of the hospitals' current standard management of PPH, as well as WHO recommendations for PPH management,[3] which included the first response steps of examination to identify the cause of bleeding, administration of uterotonics, use of intravenous fluids, and uterine massage. Providers were instructed to use UBT in cases of atonic PPH that was unresponsive to first-line measures and before recourse to surgery. In cases of retained placenta, clinicians were instructed to remove the placenta and any retained products before inserting the uterine balloon. Providers were advised to use UBT immediately upon recognition of refractory PPH and that the woman did not need to be in the operating theater to use UBT. Providers were not specifically advised to insert the balloon while women were under general anesthesia; guidance on pain management specified that women usually tolerated UBT well (this guidance based on previous experience of the Massachusetts General Hospital team) and that oral or intravenous analgesia could be used (according to local norms) if necessary. UBTs were inserted manually and without instrumentation. Each site UBT training was led by at least one master trainer who was an experienced obstetrician-gynecologist (NH, SO, SBD, ED, MCR, or AG); these master trainers had 14–30 years of clinical experience in obstetrics. Periodic supportive supervision visits were made, and refresher trainings were given on an as-needed basis.

Data collection and management

To ensure no instances of the primary outcome went undocumented, both active and passive surveillance were done (e.g. treating providers reported cases to the site coordinator, study coordinators actively solicited reports of maternal death or surgical intervention from providers, periodic review of operating room registers). Study coordinators periodically visited sites and scanned data collection forms which were then entered by one or two trained staff in each country. Scanned forms were also shared with Gynuity Health Projects in New York, who monitored data collection, cleaned the data, and merged the databases from the three study countries.

Sensitivity analysis

We conducted several *post hoc* sensitivity analyses. First, due to variations in temporal trends observed at sites, we examined whether any sites were contributing undue influence on the calculated effect sizes. The analysis of the primary outcome was repeated with each site excluded one by one to determine if the effect size changed substantially. Analysis of primary and secondary outcomes was then done excluding sites identified as outliers. A second sensitivity analysis restricted analysis to outcomes associated with postpartum hemorrhage (PPH) due to atony. Two additional sensitivity analyses were done using model extensions proposed by Hemming et al to assess interaction of temporal trends and country, and interaction of temporal trends and individual hospitals.[4] Finally, to determine if effect size estimates were sensitive to the specific statistical model used to adjust for clustering and temporal trends, we performed Poisson and negative binomial regression for primary and secondary outcomes using generalized estimating equations with robust standard errors to control for clustering by hospital (also including study phase as a covariate).

Supplementary results

Case descriptions of women who died after receiving UBT

All four women in the intervention period who died after receiving UBT had atony with no traumatic causes noted. The first woman's labor was augmented in the setting of an intra-uterine fetal demise. She developed disseminated intravascular coagulation (DIC) and lost consciousness immediately after delivering a macerated stillbirth; bleeding was unresponsive to uterotonics and the uterine balloon was inserted 30 minutes after delivery. Bleeding continued after UBT placement and the woman was transferred to another facility because the study hospital did not have blood products for managing DIC; transfer was delayed due to unavailability of the ambulance, and the woman later died in transit to the

next facility. The second woman had an uncomplicated delivery. She was discovered in the postpartum ward two hours after delivery with heavy bleeding, unconscious, and in very poor condition. Uterotonics and tranexamic acid were given immediately, but bleeding did not cease and a uterine balloon was inserted 20 minutes later. She received blood transfusion (two units whole blood, two units of fresh frozen plasma). She was transferred to the OR about 30 minutes after UBT placement, but she had cardiac arrest on the way to the OR and was resuscitated. Decision was made to transfer to a higher level facility, but she died in transit. A third woman arrived at a study facility with PPH after delivery at a lower level health center. She was diagnosed with uterine atony and retained products and received manual removal of clots. Bleeding was unresponsive to uterotonics. She was given one unit of whole blood and the uterine balloon was inserted one hour after arrival. Bleeding was noted to have slowed but did not stop. She began seizing and was administered magnesium sulphate and oxygen therapy. Blood pressure was unrecordable, adrenaline was given, a second unit of blood started, and she underwent subtotal hysterectomy 2 hours after UBT. Following the procedure, she was transferred to the post-operative ward unconscious and given a third unit of blood and later died. The fourth woman developed PPH 15 minutes after delivery. Bleeding was unresponsive to oxytocin. There was no compatible blood in the hospital blood bank, and the doctor was called but the phones were not working. The midwife decided to transfer the woman to the next level hospital and inserted the balloon at this time (approximately 25 minutes after PPH diagnosis) and bleeding stopped; however, the woman was unconscious and died before transfer, approximately 60 minutes after delivery.

References

1. Burke TF, Ahn R, Nelson BD, Hines R, Kamara J, Oguttu M, et al. A postpartum haemorrhage package with condom uterine balloon tamponade: a prospective multi-centre case series in Kenya, Sierra Leone, Senegal, and Nepal. *BJOG.* 2016 Aug; **124**(9):1532-40.

2. Burke TF, Danso-Bamfo S, Guha M, Oguttu M, Tarimo V, Nelson BD. Shock progression and survival after use of a condom uterine balloon tamponade package in women with uncontrolled postpartum hemorrhage. *International Journal of Gynecology & Obstetrics.* 2017; **139**(1): 34-8.

3. WHO. WHO recommendations for the prevention and treatment of postpartum haemorrhage. Geneva: Dept. of Reproductive Health and Research, WHO; 2012. Report No.: ISBN: 978 92 4 154850 2.

4. Hemming K, Taljaard M, Forbes A. Analysis of cluster randomised stepped wedge trials with repeated cross-sectional samples. Trials. 2017; **18**(1): 101.
